# Serum Virus-Neutralizing Antibody Titers in Clinical Trial Participants With and Without Immunocompromise Receiving Pemivibart, a Long-Acting Monoclonal Antibody for COVID-19

**DOI:** 10.1093/infdis/jiag269

**Published:** 2026-05-21

**Authors:** Kristin Narayan, Kazima Tosh, Anna Holmes, Leijun Hu, Sandeep Reddy Dkottam, Ilker Yalcin, Mark Wingertzahn

**Affiliations:** Invivyd, Inc., New Haven, Connecticut, USA; Invivyd, Inc., New Haven, Connecticut, USA; Invivyd, Inc., New Haven, Connecticut, USA; LH Pharmaceutical Consulting, LLC, Carmel, Indiana, USA; Invivyd, Inc., New Haven, Connecticut, USA; Invivyd, Inc., New Haven, Connecticut, USA; Invivyd, Inc., New Haven, Connecticut, USA

**Keywords:** COVID-19, SARS-CoV-2, monoclonal antibody, pemivibart, serum virus-neutralizing titers

## Abstract

**Background:**

Pemivibart received emergency use authorization for COVID-19 prevention in certain individuals with immunocompromise based on immunobridging calculated serum virus-neutralizing antibody (csVNA) titers. We report an analysis of measured (msVNA) titers against severe acute respiratory syndrome coronavirus 2 (SARS-CoV-2) variants before and after receipt of pemivibart in a SARS-CoV-2 immune-experienced population (CANOPY) and compare the relationship between csVNA and msVNA titers in 2 clinical trials.

**Methods:**

The trials were conducted during 2023–2024. In the phase 3 CANOPY trial, participants with (cohort A) or without (cohort B) significant immunocompromise received 2 intravenous 4500-mg pemivibart or placebo infusions 90 days apart. In the phase 1 trial, healthy adults received a single intravenous dose of pemivibart (1500, 2500, or 4500 mg). msVNA titers were determined using a SARS-CoV-2 spike pseudotyped assay; csVNA titers were calculated as serum pemivibart concentrations divided by variant-specific IC_50_ values from in vitro neutralization assays.

**Results:**

In CANOPY, predose msVNA geometric mean titers (GMTs) to the dominant circulating XBB.1.5 lineage were low across cohorts (range, 78.6–119.8), suggesting inadequate protection. msVNA titers were substantially higher against earlier Delta (B.1.617.2) and BA.4/5 variants. Pemivibart increased the day-28 msVNA GMT >55-fold against XBB.1.5, with similar levels in both cohorts. Across trials, csVNA titers were significantly correlated with msVNA titers (all Spearman correlation coefficients ≥0.73, *P* < .0001).

**Conclusions:**

These data show that previous immune experience to earlier pandemic variants provided limited cross-protection and demonstrate that pemivibart increased protective titers against newly circulating SARS-CoV-2 in individuals with and without immunocompromise. Notably, csVNA titers effectively predicted pemivibart's neutralization potential.

***Clinical Trials Registration.*** NCT06039449 and NCT05791318.

## Teaser

In clinical trials, pemivibart increased protective msVNA titers against circulating SARS-CoV-2 in individuals with and without immunocompromise. msVNA and csVNA titers were significantly correlated, supporting the use of csVNA titers as a reliable immunobridging endpoint for COVID-19 prophylaxis clinical trials.

Severe acute respiratory syndrome coronavirus 2 (SARS-CoV-2) continues to cause significant illness and mortality from COVID-19 [1–4]. Individuals with immunocompromise and adults aged ≥65 years remain at high risk for severe disease [5–7] and hospitalization [8], highlighting an ongoing need for effective COVID-19 prevention.

Serum virus-neutralizing antibody (sVNA) titers are a well-documented correlate of protection against symptomatic COVID-19 infection and severe disease [9–14] and are actively elicited by the immune system upon vaccination or infection, or passively provided via administration of a monoclonal antibody (mAb). mAbs proved to be a key modality in the effective prevention and treatment of COVID-19 during the pandemic, particularly for immune-impaired individuals [15–19]. Pemivibart is a long-acting human mAb that received emergency use authorization from the US Food and Drug Administration in March 2024 for preexposure prophylaxis of COVID-19 in certain individuals with immunocompromise based on an immunobridging endpoint of calculated sVNA (csVNA) titers in the phase 3 CANOPY trial (NCT06039449 [20]) [21]. In addition, pemivibart demonstrated strong protection from symptomatic COVID-19 (74% relative risk reduction over 12 months vs placebo) in participants without immunocompromise [17, 22], suggesting that benefit extends beyond those with immunocompromise.

csVNA titers offer important advantages over measured sVNA (msVNA) titers for accelerated clinical development of mAbs. With the rapid rate of SARS-CoV-2 evolution, the variant landscape is likely to shift before msVNA assay validation, clinical sample collection, and testing against a specific variant of interest are completed. Alternatively, csVNA titers can be determined rapidly against emerging virus variants using in vitro neutralization data and mAb concentrations (pharmacokinetic data, usually already available as a study assessment). Studies conducted prior to widespread immune experience from COVID-19 vaccination and infection demonstrated a predictive correlation between msVNA and csVNA titers or mAb concentrations [23, 24]. In these early prevention studies using mAbs, the neutralizing responses were derived exclusively from the administered mAb. Evaluation of the relationship between msVNA and csVNA titers in the post-Omicron era of COVID-19 in immune-experienced populations is important for confirming the utility of csVNA titers for immunobridging and refining titer targets for future COVID-19 mAbs [22].

In CANOPY, data from both sVNA methodologies were collected. For the primary immunobridging endpoint, csVNA titers represent the estimated neutralizing activity provided by circulating pemivibart and do not include activity provided by endogenous antibodies [21]. msVNA titers, which have not been reported previously for participants in CANOPY, represent the total neutralizing activity provided by pemivibart and any existing endogenous neutralizing activity. The evaluation of msVNA titers was a secondary endpoint assessed in serum samples using a cell-based lentiviral pseudovirus neutralization assay [25]. To confirm that csVNA titers remain predictive of msVNA titers in the setting of increasing immune exposure to SARS-CoV-2 spike, this analysis characterized msVNA titers in participants with and without immunocompromise in CANOPY before and after receipt of pemivibart. The relationship between csVNA and msVNA titers was evaluated based on data from 2 clinical trials of pemivibart, CANOPY and a phase 1 trial in healthy adults.

## METHODS

### Clinical Studies

CANOPY was conducted in the United States from September 2023 to November 2024 to evaluate the safety and efficacy of pemivibart for the prevention of COVID-19 in 2 cohorts. Cohort A was an open-label, single-arm study that enrolled participants with significant immunocompromise to receive pemivibart 4500 mg intravenously (IV) on day 1, followed by a second dose approximately 90 days later. For enrollment of cohort A, SARS-CoV-2 vaccination was allowed if it occurred ≥14 days before day 1, and prior SARS-CoV-2 infection was allowed if it occurred >120 days before day 1. Cohort B was a randomized, blinded, placebo-controlled study that enrolled adults without immunocompromise who were at risk of acquiring SARS-CoV-2 infection to receive either pemivibart or placebo (2:1) at the same dose level, 90 days apart. Prior receipt of a COVID-19 vaccine and prior SARS-CoV-2 infection were allowed if they occurred >120 days before randomization. Details of CANOPY, including the methods, primary immunobridging endpoint, and interim safety and efficacy, have been described [17, 21]. The evaluation of msVNA titers against relevant SARS-CoV-2 variants after receipt of pemivibart was a secondary objective.

The single ascending-dose phase 1 trial (NCT05791318 [26]) was conducted from March 2023 to May 2024 to investigate the safety, tolerability, and pharmacokinetics of 3 doses of pemivibart (1500, 2500, and 4500 mg) in healthy adults [26–28].

The trials were conducted in accordance with the principles of the Declaration of Helsinki, and all participants provided written informed consent.

### Sample Collection

In CANOPY, blood samples for msVNA and pemivibart pharmacokinetic assessments were collected at day 1 (predose for sVNA; postdose for PK), day 28, and months 3, 6, and 12, with an additional msVNA sample collected on day 14 for cohort A. In the phase 1 trial, samples for msVNA were collected at day 1 (predose), days 7, 14, 21, and 45, and months 3, 6, and 12. Samples at the same time points were evaluated for csVNA titers, except for pharmacokinetic data from the day 1 predose sample, in which no pemivibart was detected.

### msVNA Titer Assay

msVNA titers were assessed against 3 SARS-CoV-2 variants (XBB.1.5, BA.4/5, Delta [B.1.617.2]) using a lentivirus-based pseudovirus assay with firefly luciferase reporter (PhenoSense SARS-CoV-2 NAb assay; Labcorp-Monogram Biosciences, South San Francisco, CA) [25]. msVNA measures were performed as a dilution series in singlet. Briefly, test sera were serially diluted starting at 1:40 and covering 10 three-fold dilutions. The serum dilutions were incubated with pseudovirus for 1–2 hours at 37°C and then incubated with HEK-293 target cells expressing hACE2 and TMPRSS2 for 3 days at 37°C. Pseudovirus infection inhibition was measured by luciferase activity. The reciprocal of the serum dilution corresponding to 50% inhibition of virus infection was reported. Reported titer values below the limit of detection (<40) or samples demonstrating nonspecific activity to a non-SARS-CoV-2 specificity control pseudovirus were imputed to a titer of 20 for analysis purposes. sVNA titers were excluded from analysis if they were collected from participants who had postdose SARS-CoV-2 vaccination, polymerase chain reaction (RT-PCR)-confirmed SARS-CoV-2 infection, seroconversion to N-antigen positive, or administration of an mAb (other than investigational pemivibart).

### csVNA Titer Determination

Estimated csVNA titers were determined by dividing the serum concentration of pemivibart with the in vitro half maximal inhibitory concentration (IC_50_) of neutralizing activity against 3 SARS-CoV-2 variants (XBB.1.5, BA.4/5, Delta) and expressed as 1/dilution for analysis. Serum concentrations of pemivibart were assessed using a validated electrochemiluminescence method at PPD® Laboratories (Richmond, VA). The in vitro neutralizing activity of pemivibart against SARS-CoV-2 variants was performed at Monogram Biosciences, as described previously [17, 21].

### Serology Testing

The Roche (Indianapolis, IN) Elecsys anti-SARS-CoV-2 and anti-SARS-CoV-2 S assays were used to determine antinucleoprotein (N) antibodies against SARS-CoV-2 and to measure receptor-binding domain-targeting antibodies (S-protein status) at baseline, respectively. Testing was performed using validated assays at PPD GCL (Highland Heights, KY).

### Statistical Analysis

Study populations are described in Table 1. For sVNA titers, geometric mean titers (GMT) and geometric mean fold rise (GMFR) from day 1 predose were summarized by time point in both cohorts and by treatment group in cohort B, and 95% confidence intervals (CIs) were computed as applicable. GMFR is the geometric mean of the ratios of postdose titer to baseline titer.

**Table 1.** CANOPY Study Populations

Assessment/Endpoint	Population	
	Cohort A (Participants with Immunocompromise)	Cohort B (Participants without Immunocompromise)
Baseline characteristics	SAS, all participants who received any amount of study drug	FAS, all randomized participants
msVNA titers	FAS, all participants who received a full initial dose of pemivibart mFAS, a subset of participants in the FAS with undetectable or nonspecific serum-neutralizing activity (msVNA < 40) at baseline	mFAS, participants without current SARS-CoV-2 infection at baseline (confirmed by central laboratory RT-PCR) who received any amount of pemivibart or placebo
csVNA titers	PK-FAS, participants who received the full initial dose and had a quantifiable pemivibart serum concentration on day 28	

Abbreviations: FAS, full analysis set; mFAS, modified full analysis set; msVNA, measured serum virus-neutralizing antibody; PK-FAS, pharmacokinetic full analysis set; SAS, safety analysis set.

Repeated-measures mixed-model analysis was performed to assess the relationship between msVNA and csVNA titers for samples in which data were available for both assessments. Data for cohorts A and B in CANOPY were combined for analysis. msVNA and csVNA titers were log_10_-transformed for analysis. A random intercept-only model was used to account for differences in endogenous titers across participants. To quantify within-subject correlation while controlling for between-subject variability, repeated-measures correlation to ranked log_10_-transformed values was used, yielding Spearman-type correlation coefficients [29]. Two-sided *P* values <.05 were considered statistically significant. Statistical analyses were performed using SAS Proc CORR (Correlation procedure) version 9.4 (SAS Institute, Cary, NC, USA).

## RESULTS

### Baseline COVID-19 Immunology

Participants in CANOPY received an initial dose of pemivibart between 8 September 2023, and 8 November 2023, when variants related to the XBB.1.5 lineage, including EG.5, were the dominant variants in circulation and the XBB.1.5 spike sequence was the source of the monovalent COVID-19 booster. As reported previously [17], all participants in cohort A had significant immune compromise (Supplementary Table 1) and 87.9% (269/306) had been vaccinated previously, having received a median of 5 COVID-19 vaccinations prior to enrollment (Table 2). The majority (60.8%) received COVID-19 vaccination within 1 year prior to initial dose of pemivibart, and 46 (15.0%) received the XBB.1.5 booster prior to enrollment. At baseline, 16.7% of participants reported prior COVID-19 infection based on medical history. Almost all participants in cohort A (97.7%) were positive for antibodies to the SARS-CoV-2 S-protein, consistent with previous exposure to infection or vaccination, and 49.0% were positive for antibodies to the N-protein, elicited specifically by previous infection (Table 2).

**Table 2.** CANOPY Baseline COVID-19 Immunology Characteristics

Baseline Characteristic^a^	Cohort A^b^ (N = 306)	Cohort B^c^ Pemivibart (n = 320)	Cohort B^c^ Placebo (n = 160)	Cohort B^c^ Overall (N = 480)
Participants with prior vaccination				
Total	269 (87.9)	192 (60.0)	100 (62.5)	292 (60.8)
Within 1 year prior to first ppt dosed^d^	186 (60.8)	55 (17.2)	40 (25.0)	95 (19.8)
Within 6 months prior to first ppt dosed^d^	116 (37.9)	5 (1.6)	1 (0.6)	6 (1.3)
Received fall 2023 XBB booster^e^	46 (15.0)	0	0	0
Number of prior COVID-19 vaccinations				
Median (min, max)	5.0 (1.0, 11.0)	3.0 (1.0, 6.0)	3.0 (1.0, 5.0)	3.0 (1.0, 6.0)
Participants with medical history of COVID-19 infection	51 (16.7)	62 (19.4)	37 (23.1)	99 (20.6)
Baseline serology S-protein status				
Positive	299 (97.7)	315 (98.4)	159 (99.4)	474 (98.8)
Negative	2 (0.7)	2 (0.6)	1 (0.6)	3 (0.6)
Missing	5 (1.6)	3 (0.9)	0	3 (0.6)
Baseline serology N-protein status				
Positive	150 (49.0)	271 (84.7)	137 (85.6)	408 (85.0)
Negative	151 (49.3)	46 (14.4)	23 (14.4)	69 (14.4)
Missing	5 (1.6)	3 (0.9)	0	3 (0.6)
Baseline measured XBB.1.5 sVNA titer below detection, n (%)	n = 306	n = 135^f^	n = 81^f^	n = 216^f^
<40	174 (56.9)	86 (63.7)	43 (53.1)	129 (59.7)
≥40	126 (41.2)	51 (37.8)	39 (48.1)	90 (41.7)
Missing	6 (2.0)	183 (nt)	78 (nt)	261 (nt)

Abbreviations: N, nucleocapsid; Nt, not tested; ppt, participant; S, spike.

^a^Data represent no. (%) of participants unless otherwise specified.

^b^Cohort A is based on safety analysis set, which included all participants who received any amount of pemivibart.

^c^Cohort B is based on full analysis set (FAS), which included all randomized participants. Note that after the 6-month data were reported in the interim analysis [17], 2 participants were identified as having been enrolled in the trial twice at different sites (each randomized to both pemivibart and placebo); these 2 participants were excluded from the FAS.

^d^Calculated from cutoff first participant dosed–15 September 2023 (cohort A); 8 September 2023 (cohort B).

^e^Calculated from first 2023 booster approval–11 September 2023.

^f^In cohort B, analyses of msVNA titers were performed in the modified FAS (mFAS), defined as participants without current SARS-CoV-2 infection at baseline (confirmed by central laboratory real-time polymerase chain reaction) and who received any amount of pemivibart or placebo. Due to issues with sample processing for the neutralization assay, the samples from the first 200 participants could not be used. Therefore, an additional 216 participants were subsequently chosen based on sample availability to assess msVNA titers.

Overall, fewer participants in cohort B (60.8% [292/480]) had been vaccinated previously versus cohort A, having received a median of 3 COVID-19 vaccinations prior to enrollment (Table 2). Only 19.8% of cohort B had received a COVID-19 vaccine within 1 year before the start of dosing (per protocol). At baseline, 20.6% of participants reported prior COVID-19 infection based on medical history. Almost all participants in cohort B (98.8%) were positive for antibodies to the SARS-CoV-2 S-protein, and a higher percentage (85.0%) were positive for antibodies to the N-protein versus cohort A, possibly due to more recent COVID-19 infections, more robust antibody response to infection, or more N seroconversion related to less vaccination in this cohort [30]. Baseline COVID-19 immune characteristics were balanced across pemivibart and placebo arms in cohort B (Table 2).

In the phase 1 study, all participants (N = 30, 100%) were positive for antibodies to the SARS-CoV-2 S-protein and 24/30 (80%) were positive for antibodies to the N-protein at baseline (Supplementary Table 2).

### msVNA and csVNA Titers in Participants With Immunocompromise (Cohort A)

Samples from 298 cohort A participants were available to evaluate msVNA titers. At baseline, more than half (56.9%) had undetectable or nonspecific serum-neutralizing activity against XBB.1.5 (msVNA < 40) (Table 2) and, overall, the day 1 predose msVNA GMT to XBB.1.5 was low (114.3 [95% CI, 88.2–148.2]) (Table 3). Participants who received an XBB.1.5 monovalent vaccine prior to enrollment (n = 45) demonstrated higher predose msVNA GMTs to XBB.1.5 (668.2 [95% CI, 332.4–1343.3]) than those who did not (n = 253; 82.9 [63.8–107.71]) (Supplementary Table 3).

**Table 3.** CANOPY Cohort A—Summary of Measured and Calculated sVNA Titers Against XBB.1.5

	Measured SVNA (msVNA)				Calculated SVNA (csVNA)
	FAS (n = 298)		mFAS (n = 164)		PK-FAS (n = 289)
Study Day	GMT (95% CI)	GMFR From Day 1 (95% CI)	GMT (95% CI)	GMFR From Day 1 (95% CI)	GMT (95% CI)
Day 1 (predose)	114.3 (88.2–148.2)	N/A	20.0 (N/A)	N/A	N/A
Day 14	9290.5 (8090.8–10 668.1)	79.1 (60.2–104.0)	7708.2 (6131.1–9691.1)	385.4 (306.6–484.6)	5828.1 (5593.2–6072.8)
Day 28	6460.2 (5604.7–7446.2)	56.8 (43.6–74.1)	5116.9 (4107.3–6374.8)	255.8 (205.4–318.7)	4490.6 (4333.0–4653.8)
Month 3 (predose)	2595.9 (2175.7–3097.3)	N/A	1963.5 (1510.0–2553.2)	N/A	2030.7 (1927.0–2140.0)
Month 6	2352.1 (1883.4–2937.3)	N/A	1441.3 (1020.2–2036.3)	N/A	2552.1 (2435.0–2674.9)
Month 12	329.5 (229.4–473.3)	N/A	146.3 (91.0–235.3)	N/A	123.9 (114.1–134.5)

Abbreviations: FAS, full analysis set; GMFR, geometric mean fold rise; GMT, geometric mean titer; mFAS, modified full analysis set; N/A, not applicable; PK-FAS, pharmacokinetic full analysis set; SAS, safety analysis set.

A single dose of pemivibart increased msVNA GMTs to XBB.1.5 (GMFR: 79.1 and 56.8 at days 14 and 28, respectively) in the full analysis set (FAS) (Table 3). msVNA GMTs against XBB.1.5 were comparable at month 3 prior to redosing (2595.9) and month 6 after redosing (2352.1) (Table 3). In the subset of participants with undetectable or nonspecific serum-neutralizing activity against XBB.1.5 at baseline (msVNA < 40), defined as the modified FAS (mFAS) for cohort A (n = 164), msVNA titers increased substantially at day 28 following initial pemivibart dosing to levels similar but slightly lower than those achieved in the FAS (GMTs 5116.9 and 6460.2, respectively, Table 3), likely due to lack of preexisting neutralizing antibodies to XBB.1.5. Overall, msVNA GMTs to XBB.1.5 remained above day 1 predose levels through month 12 in the FAS and mFAS (Table 3).

Overall, csVNA and msVNA titers were comparable (<2-fold difference) at time points within 1–6 months post-pemivibart dosing, with more variation observed following pemivibart washout by month 12, as an increasing proportion of measured neutralizing activity is contributed by endogenous antibodies (Table 3).

Predose msVNA titers against older variants (BA.4/5 and Delta) were substantially higher than those against XBB.1.5 (Table 4), consistent with previous vaccinations and prior immune experience to variants that were prevalent at earlier stages of the pandemic. In the FAS, pemivibart increased the day-28 msVNA against BA.4/5 and Delta with GMFRs of 32.0 and 90.8, respectively (Table 4). High titers observed postdosing are consistent with relatively more potent pemivibart neutralization of BA.4/5 (IC_50_, 47.9 ng/mL) and Delta (IC_50_, 7.6 ng/mL) versus XBB.1.5 (IC_50_, 104.3 ng/mL).

**Table 4.** CANOPY Cohort A—Summary of msVNA Titers Against BA.4/5 and Delta

	MsVNA (FAS)			
	BA.4/5 (n = 298)		Delta (n = 298)	
Study Day	GMT (95% CI)	GMFR From Day 1 (95% CI)	GMT (95% CI)	GMFR From Day 1 (95% CI)
Day 1 (predose)	638.2 (475.2–857.2)	N/A	2217.1 (1699.3–2892.5)	N/A
Day 14	27 864.8 (25 885.1–29 995.9)	42.3 (31.9–56.1)	262 159.5 (243 324.6–282 452.2)	115.0 (87.6–150.9)
Day 28	20 618.2 (18 817.1–22 591.6)	32.0 (24.3–42.1)	205 029.8 (191 186.5–219 875.5)	90.8 (69.8–118.0)
Month 3 (predose)	9088.4 (7725.2–10 692.1)	N/A	82 058.0 (75 474.3–89 216.0)	N/A
Month 6	7860.1 (6372.1–9695.5)	N/A	92 065.4 (81 402.5–104 125.2)	N/A
Month 12	1262.2 (894.3–1781.4)	N/A	5774.2 (4088.7–8154.7)	N/A

Abbreviations: FAS, full analysis set; GMFR, geometric mean fold rise; GMT, geometric mean titer; msVNA, measured serum virus-neutralizing antibody; N/A, not applicable.

### msVNA Titers in Participants Without Immunocompromise (Cohort B)

Based on availability, 216 samples from consecutively enrolled cohort B participants (135 pemivibart and 81 placebo) were selected to evaluate msVNA titers (Table 1). Similar to cohort A, the majority of cohort B mFAS participants had undetectable or no specific neutralizing activity against XBB.1.5 at baseline (pemivibart arm: 86/135 [63.7%]; placebo arm: 43/81 [53.1%]) (Table 2). In addition, day 1 predose msVNA titers against XBB.1.5 were low (GMT: 78.6 and 119.8 for the pemivibart and placebo arms, respectively) (Table 5). Consistent with previous infection or vaccination with earlier variants, day 1 predose msVNA titers against BA.4/5 or Delta were substantially higher than those against XBB.1.5. Pemivibart increased msVNA GMTs in cohort B similar to levels in the cohort A FAS across all variants evaluated (Table 5 and Figure 1). Following redosing at month 3, msVNA GMTs in the pemivibart arm remained above day 1 predose levels through month 12. In the placebo arm, GMTs were generally unmodulated over the course of 12 months against all variants analyzed (Table 5 and Figure 1).

**Figure 1.**
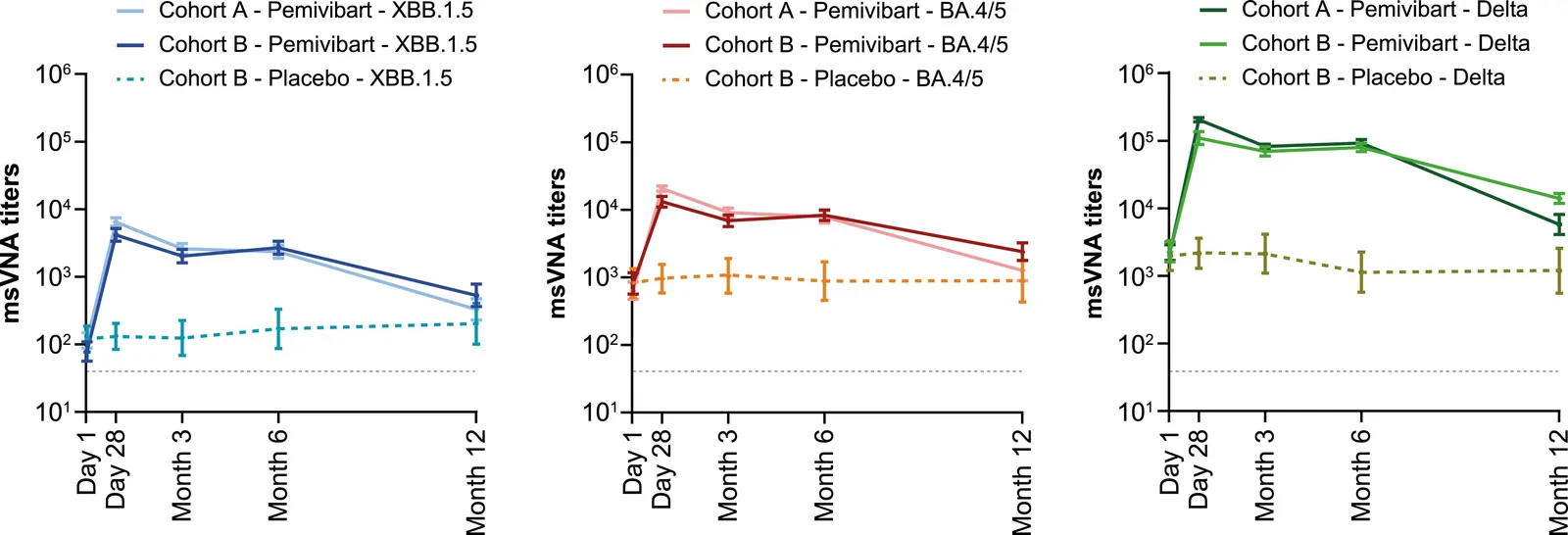
Summary of msVNA titers against 3 SARS-CoV-2 variant pseudovirus in individuals with (cohort A) and without (cohort B) immunocompromise from CANOPY. msVNA GMTs over time against XBB.1.5, BA.4/5, and Delta pseudoviruses are presented for cohort A FAS and cohort B mFAS, including pemivibart and placebo arms, as indicated in the legend. The starting dilution or lower limit of detection for neutralization (40) is indicated by a dotted horizontal black line. Error bars represent GMT 95% CI at each time point. Additional details are available in Tables 3–5.

**Table 5.** CANOPY Cohort B—Summary of msVNA Titers Against XBB.1.5, BA.4/5, and Delta by Study Treatment

Study Day	Pemivibart (mFAS) (n = 135)		Placebo (mFAS) (n = 81)	
	GMT (95% CI)	GMFR From Day 1 (95% CI)	GMT (95% CI)	GMFR From Day 1 (95% CI)
*XBB.1.5*				
Day 1 (predose)	78.6 (56.7–109.0)	N/A	119.8 (77.0–186.4)	N/A
Day 28	4174.1 (3359.3–5186.5)	63.0 (44.1–89.9)	131 (84.2–203.9)	1.1 (0.8–1.5)
Month 3 (predose)	2026.6 (1612.1–2547.8)	25.4 (17.2–37.4)	124.3 (68.5–225.6)	1.0 (0.6–1.5)
Month 6	2692.9 (2160.3–3357.0)	30.4 (19.7–46.9)	169.3 (86.7–331.0)	1.4 (0.8–2.6)
Month 12	531.6 (363.6–777.1)	5.6 (3.6–8.8)	202.9 (100.7–408.8)	1.5 (0.8–2.6)
*BA.4/5*				
Day 1 (predose)	812.3 (565.6–1166.6)	N/A	824.5 (503.6–1349.9)	N/A
Day 28	13 186.4 (10 995.6–15 813.8)	18.5 (13.2–25.9)	953.4 (585.3–1553.3)	1.1 (0.9–1.4)
Month 3 (predose)	6888.2 (5663.6–8377.6)	10.4 (6.9–15.5)	1051.1 (583.3–1893.8)	1.3 (1.0–1.9)
Month 6	8288.3 (6929.7–9913.2)	11.5 (7.7–17.4)	879.4 (456.4–1694.6)	1.4 (0.9–2.2)
Month 12	2399.5 (1782.3–3230.6)	3.0 (2.1–4.5)	872.2 (430.3–1767.9)	1.1 (0.7–1.9)
*Delta*				
Day 1 (predose)	2268.9 (1613.8–3190.0)	N/A	2002.5 (1203.0–3333.3)	N/A
Day 28	109 405.3 (87 514.8–136 771.3)	58.6 (41.5–82.8)	2156.6 (1293.1–3596.8)	1.1 (0.8–1.5)
Month 3 (predose)	69 481.8 (59 530.7–81 096.4)	37.2 (25.3–54.8)	2130.1 (1098.1–4132.0)	1.3 (0.9–1.9)
Month 6	79 391.9 (69 038.5–91 298.0)	42.5 (27.7–65.0)	1138.6 (573.9–2259.0)	0.9 (0.6–1.3)
Month 12	13 972.9 (11 817.4–16 521.7)	6.5 (4.5–9.4)	1192.3 (554.7–2563.0)	0.7 (0.5–0.9)

Abbreviations: GMFR, geometric mean fold rise; GMT, geometric mean titer; mFAS, modified full analysis set; N/A, not applicable.

### Correlation of Measured and Calculated sVNA Titers

In 2 clinical trials, msVNA titers were significantly correlated with csVNA titers for each variant tested, with Spearman correlation coefficients against XBB.1.5, BA.4/5, and Delta, respectively, of ρ = 0.81, 0.79, and 0.89 (all *P* < .0001) in the phase 1 trial (Figure 2*A*), and ρ = 0.73, 0.80, and 0.88 (all *P* < .0001) in CANOPY (both cohorts combined, Figure 2*B*). More variability in the correlative relationship was observed at later time points (eg, month 12), as pemivibart concentrations waned and a higher proportion of the msVNA titers was due to endogenous sVNA activity.

**Figure 2.**
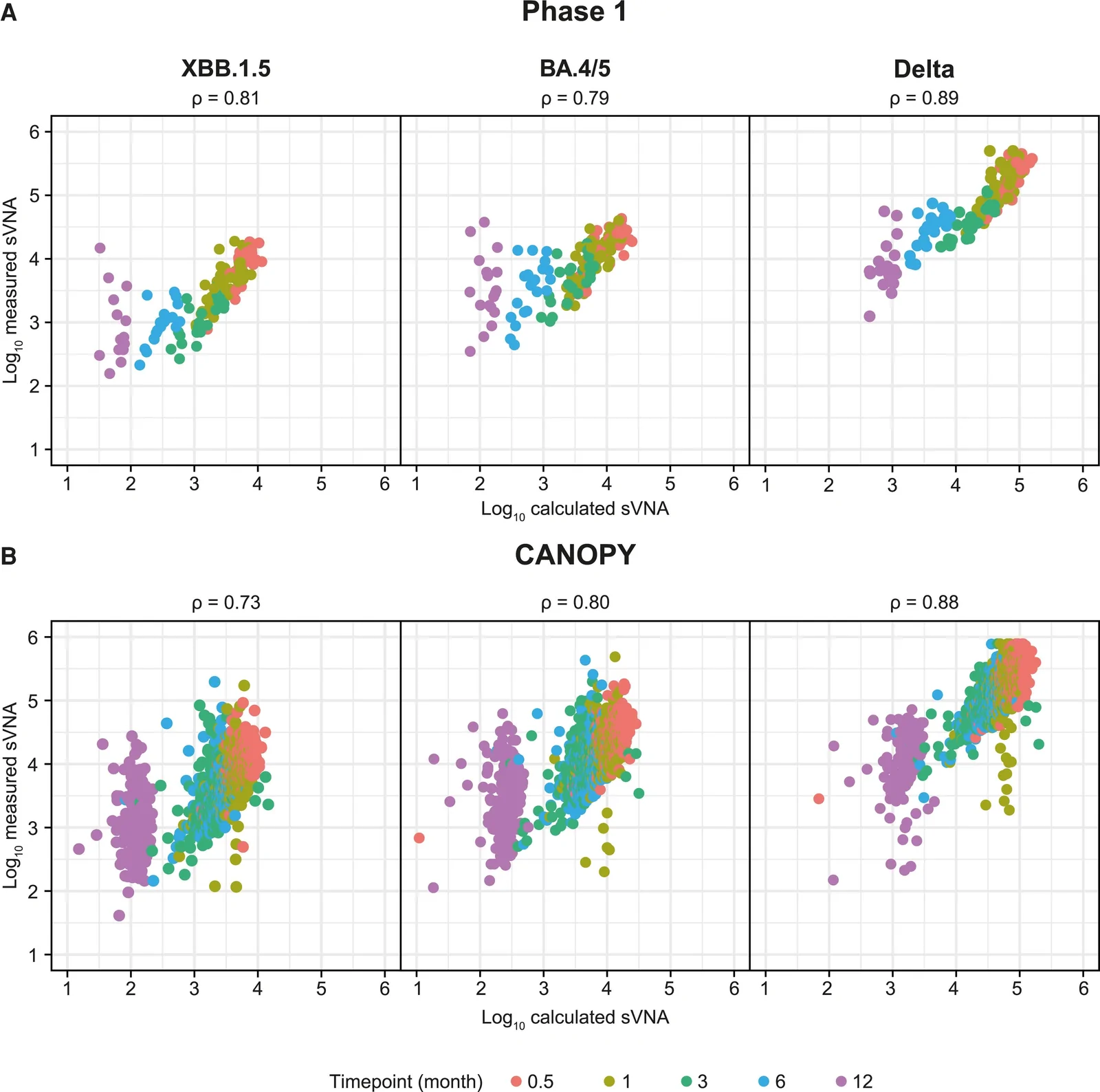
Correlation between msVNA and csVNA titers for (*A*) phase 1 trial and (*B*) CANOPY. Data points represent log_10_-transformed msVNA and csVNA titer data from 1 serum sample and are color coded based on time of sample collection. For phase 1, available PK and msVNA data from days 7 and 14 (labeled as month 0.5), days 21 and 45 (labeled as month 1), and months 3, 6, and 12 time points were analyzed. For CANOPY, available PK and msVNA data from day 14 (labeled as month 0.5), day 28 (labeled as month 1), and months 3, 6, and 12 time points were analyzed. Day 1 data and titers below the limit of quantification were excluded from the analysis. All *P* values were <.0001.

## DISCUSSION

Pemivibart received emergency use authorization for the preexposure prevention of COVID-19 in certain individuals with immunocompromise in March 2024 based on immunobridging csVNA titers against JN.1 (the relevant circulating variant at the time) to sVNA titers of historical COVID-19 mAbs associated with protection [21, 31]. Pemivibart continues to maintain in vitro neutralization potency against recent circulating omicron sublineages, including KP.3.1.1, XEC, LP.8.1, and XFG [32]. This analysis evaluated whether csVNA titers remain predictive of msVNA titers, in the setting of increasing immune exposure to SARS-CoV-2 in the population. Data from the CANOPY trial demonstrate that a substantial proportion of participants (>57%) had undetectable msVNA titers against a dominant circulating XBB.1.5 lineage at baseline, and that pemivibart substantially increased protective msVNA titers against XBB.1.5 in individuals with and without immunocompromise. Furthermore, a strong correlation was observed between msVNA and csVNA titers across 2 clinical trials. These data support previous studies across COVID-19 mAb development programs showing that msVNA titers correlate well with csVNA titers [23, 24]. csVNA offers an established, standalone titer value to effectively predict neutralization potential of mAbs.

Participants in CANOPY represent a SARS-CoV-2 immune-experienced population, as >98% were positive for antibodies to the SARS-CoV-2 S-protein at baseline. Despite multiple previous vaccinations and prior immune experience, baseline msVNA titers against XBB.1.5, the contemporary circulating lineage at the start of CANOPY, were low in participants with and without immunocompromise (GMT range, 78.6–119.8). Although higher predose msVNA titers were observed in individuals with immunocompromise who received the XBB.1.5 monovalent vaccine prior to enrollment than in those who did not (668.2 vs 82.9), a titer of 668.2 is lower than XBB.1.5 monovalent vaccine titers previously observed in healthy participants [33]. The msVNA titers to XBB.1.5 were also well below the target immunobridging threshold for pemivibart (3514) [21] across both cohorts, suggesting inadequate protective titers at baseline in study participants, regardless of immune system status.

In contrast, baseline msVNA titers were higher against earlier variants (BA.4/5 and Delta) in both cohorts, suggesting that previous infections or vaccinations based on older variants provided limited cross-protection against more recent circulating variants. Moreover, the data indicate the possibility that immune imprinting from previous infections or vaccines derived from ancestral SARS-CoV-2 variants may act to limit the responses needed to control infections caused by modern circulating lineages [34–36]. Finally, predose neutralizing titers against 3 distinct SARS-CoV-2 lineages were generally similar in participants with and without immunocompromise. Combined with the clinical efficacy demonstrated for pemivibart in cohort B [17], the msVNA profiles support a benefit of mAb prophylaxis that extends beyond people who have immunocompromise to other individuals who are highly susceptible and at risk.

Data from CANOPY show that one 4500 mg dose of pemivibart increased msVNA GMTs against XBB.1.5 > 55-fold by day 28 postdose in both cohorts. The pemivibart msVNA titers surpassed that elicited by XBB.1.5 monovalent booster immunization. Chalkias et al [33] reported a GMFR against XBB.1.5 of 16.7 (predose GMT: 155; day 15 GMT: 2579) in participants without immunocompromise receiving XBB.1.5 monovalent booster as a fifth vaccine dose, using a comparable lentivirus pseudovirus reporter virus assay readout. While assay variations across labs could result in differences in absolute titers, evaluation of the fold rise in sVNA titers observed within an individual study offers a more controlled assessment to compare vaccine or mAb benefit. Notably, the predose GMTs reported by Chalkias et al were also low against the contemporary variant XBB.1.5 despite multiple previous vaccinations, similar to observations in both cohorts in CANOPY [33]. Using a vesicular stomatitis virus–pseudotyped virus assay, Wang et al [37] showed that vaccination with the XBB.1.5 monovalent booster resulted in a 27-fold increase in XBB.1.5-directed neutralizing titers (predose GMT: 21; postdose GMT: 561) in previously vaccinated healthy donors without recent XBB.1.5 infection and a 9.5-fold increase among those with recent XBB.1.5 infection (predose GMT: 541; postdose GMT: 5125), with an average of 26 days between prevaccine and postvaccine serum collection. A high degree of variability in immune response is evident with vaccine boosters given differences in immune responses across individuals. In CANOPY, mAb prophylaxis led to predictable sVNA responses and consistent titer levels across recipients, regardless of immune function or previous immune history.

There are limitations of this analysis. First, the current study was limited to a single mAb, pemivibart, which may limit the generalizability of the results to other mAbs. Second, these data characterize msVNA titers against 3 previously circulating variants (Delta, BA.4/5, and XBB.1.5); preexisting titer levels and profiles of participants with and without immunocompromise could be different against more modern Omicron sublineages (eg, LP.8.1, XFG). Also, baseline COVID-19 immunological characteristics, including numbers of vaccinations and serostatus, may vary if analyzed in the current year, given additional immune exposures. While pemivibart shows continued activity against current SARS-CoV-2 circulating variants [32], the variant landscape changes quickly and ongoing analyses of circulating variants must be conducted to ensure predictability of the neutralization potential of pemivibart.

COVID-19 continues to cause sickness and death worldwide [1, 4]. Despite the availability of vaccines and the high proportion of SARS-CoV-2-seropositive individuals in the general population, immunity levels remain heterogeneous [38]. Recent data in people who were critically ill with the Omicron variant show that low sVNA titers on admission are a predictor of day 28 mortality, irrespective of immunosuppression status [9]. mAbs can provide an additional option for the prevention of COVID-19 by maintaining serum neutralization activity against contemporary variants in individuals with or without immunocompromise. Neutralizing activity can be monitored as new variants emerge, such that physicians are informed of the suitability and continued activity of an mAb in advance of a surge, rather than learning retrospectively of efficacy in postsurge epidemiology or surveillance reports, as is commonly the case for vaccines against novel variants. Finally, the large body of evidence establishing sVNA titers as a surrogate of clinical protection supports continued use of immunobridging sVNA titers as an accelerated path to develop novel mAbs for the fight against SARS-CoV-2.

## Supplementary Material

jiag269_Supplementary_Data

## Supplementary Data

Supplementary materials are available at *The Journal of Infectious Diseases* online (http://jid.oxfordjournals.org/). Supplementary materials consist of data provided by the author that are published to benefit the reader. The posted materials are not copyedited. The contents of all supplementary data are the sole responsibility of the authors. Questions or messages regarding errors should be addressed to the author.
