# Supplementary material for: Serum Virus-Neutralizing Antibody Titers in Clinical Trial Participants With and Without Immunocompromise Receiving Pemivibart, a Long-Acting Monoclonal Antibody for COVID-19
Source: J Infect Dis. 2026 May 21;234(4):e859–68. doi: 10.1093/infdis/jiag269 (PMC13668337; doi:10.1093/infdis/jiag269)
Supplement: Supplementary file 1 [file jiag269_supplementary_data.docx]

# **SUPPLEMENT**

Serum Virus-Neutralizing Antibody Titers in Clinical Trial Participants With and Without Immunocompromise Receiving Pemivibart, a Long-Acting Monoclonal Antibody for COVID-19

## **Supplementary Table 1. CANOPY Cohort A – Summary of immunocompromise conditions^1^**

| Immunocompromise condition^a^ | Cohort A  (n = 306) |
| --- | --- |
| Moderate/severe primary immunodeficiency | 37 (12.1) |
| Acute leukemia, CLL, NHL, multiple myeloma | 40 (13.1) |
| Actively treated solid tumor or hematologic cancer | 20 (6.5) |
| SOT recipient receiving immunosuppressive therapy | 33 (10.8) |
| Other immunosuppressive medications^b^ | 202 (66.0) |
| Advanced HIV (CD4 cell count <350/µL) | 27 (8.8) |

^a^Participants may have >1 immunocompromising condition or medication.

^b^Taking high-dose corticosteroids (≥20 mg of prednisone or equivalent per day for ≥2 weeks), B-cell–depleting agents (within the past year), alkylating agents, antimetabolites, transplant-related immunosuppressive drugs, tumor necrosis factor blockers, or other immunosuppressive or immunomodulatory biologic agents.

Abbreviations: CLL, chronic lymphocytic leukemia; HIV, human immunodeficiency virus; NHL, non-Hodgkin lymphoma; SOT, solid organ transplant.

1. Wolfe CR, Cohen J, Mahoney K, et al. Safety and efficacy of pemivibart, a long-acting monoclonal antibody, for prevention of symptomatic COVID-19: interim results from the CANOPY clinical trial. *Clin Infect Dis* 2025;81:439-50.

**Supplementary Table 2. Phase 1 Study – Summary of Baseline Characteristics**

| Characteristic | Pemivibart  (n = 24) | Placebo  (n = 6) | Total  (n = 30) |
| --- | --- | --- | --- |
| Age, years, mean (range) | 34.5 (20‒62) | 36.7 (24‒58) | 35.0 (20‒62) |
| Sex, n (%) |  |  |  |
| Female | 10 (41.7) | 4 (66.7) | 14 (46.7) |
| Male | 14 (58.3) | 2 (33.3) | 16 (53.3) |
| Race, n (%) |  |  |  |
| Asian | 3 (12.5) | 0 | 3 (10.0) |
| Black/African American | 1 (4.2) | 0 | 1 (3.3) |
| White | 20 (83.3) | 5 (83.3) | 25 (83.3) |
| Other | 0 | 1 (16.7) | 1 (3.3) |
| Positive serology S-protein, n (%) | 24 (100) | 6 (100) | 30 (100) |
| Positive serology N-protein, n (%) | 19 (79.2) | 5 (83.3) | 24 (80.0) |

Abbreviations: N, nucleocapsid; S, spike.

## **Supplementary Table 3. CANOPY Cohort A – Summary of Measured sVNA Titers by XBB.1.5 Vaccination Status**

|  | Measured sVNA against XBB.1.5 | | | |
| --- | --- | --- | --- | --- |
| Study day | Received fall 2023 XBB booster (n = 45) | | Did not receive fall 2023 XBB booster (n = 253) | |
|  | GMT (95% CI) | GMFR from day 1 (95% CI) | GMT (95% CI) | GMFR from day 1 (95% CI) |
| Day 1 (predose) | 668.2  (332.4–1343.3) | N/A | 82.9  (63.8–107.7) | N/A |
| Day 14 | 11669.8  (9295.0–14 651.4) | 17.5  (8.8–34.6) | 8907.9  (7604.0–10 435.3) | 105.1  (78.9–139.9) |
| Day 28 | 7461.8  (5423.2–10 266.7) | 11.2  (5.5–22.6) | 6285.7  (5364.6–7365.1) | 77.8  (59.5–101.9) |

Abbreviations: CI, confidence interval; GMFR, geometric mean fold-rise; GMT, geometric mean titer; N/A, not applicable; sVNA, serum virus-neutralizing antibody.
